# The prognostic value of muscle regional oxygen saturation index in severe community-acquired pneumonia: a prospective observational study

**DOI:** 10.1186/s40560-016-0129-4

**Published:** 2016-01-19

**Authors:** Laura Claverias, Michael Marí, Judith Marín-Corral, Mónica Magret, Sandra Trefler, María Bodí, Antonio García-España, Juan Carlos Yébenes, Sergi Pascual, Joaquim Gea, Alejandro Rodríguez

**Affiliations:** Joan XXIII University Hospital, Critical Care Department, IISPV/URV, Carrer Dr. Mallafre Guasch 4, 43007, Tarragona, Spain; Research Unit, Joan XXIII University Hospital, IISPV/URV, Tarragona, Spain; CIBER de enfermedades respiratorias (CIBERES), ISC III, Bunyola Palma de Mallorca, Spain; Parc de Salut Mar, IMIM, Pneumology Department, CEXS, UPF, Barcelona, Spain; Critical Care Department, Mataró Hospital, Mataró, Spain

**Keywords:** Community-acquired pneumonia, Sepsis, Septic shock, Microcirculation, Near-infrared spectroscopy

## Abstract

**Background:**

Community-acquired pneumonia (CAP) mortality exceeds 20 % in critical care patients despite appropriate antibiotic therapy. Regional tissue oxygen saturation index (rSO2) measured with near-infrared spectroscopy (NIRS) might facilitate early detection for patients at risk of serious complications. Our objectives were to determine the relationship between early determination of rSO2 and mortality and to compare discrimination power for mortality of rSO2 and other resuscitation variables in critically ill CAP patients.

**Methods:**

This is a prospective observational study. Patients with CAP were enrolled within 6 h to intensive care admission. Demographics and clinical variables were recorded. rSO2 was determined using NIRS in brachioradialis muscle. All variables were determined at baseline and 24 h after admission.

**Results:**

Forty patients were enrolled. Fourteen patients (35 %) had a baseline rSO2 < 60 % and 7 of them died (50 %). Only 1 of 26 (3.8 %) patients with rSO2 ≥ 60 % died (*p* = 0.007). The area under ROC curve (AUROC) showed consistent mortality discrimination at baseline (0.84, *p* = 0.03) and at 24 h (0.86, *p* = 0.006) for rSO2 values. Cox regression analysis showed that “low” rSO2 at ICU admission (hazard ratio (HR) = 8.99; 95 % confidence interval (CI) 1.05–76.8; *p* = 0.045) and “low” rSO2 at 24 h (HR = 13.18; 95 % CI 1.52–113.6; *p* = 0.019) were variables independently associated with mortality. In contrast, other variables such as Acute Physiology and Chronic Health Evaluation (APACHE II) score (HR = 1.09; 95 % CI 0.99–1.19; *p* = 0.052) were not associated with mortality.

**Conclusions:**

Our findings suggest that forearm skeletal muscle rSO2 differs in patients with severe CAP according to outcome and might be an early prognosis tool.

## Background

Community-acquired pneumonia (CAP) is an important cause of morbidity, mortality and increased health-care costs [1–3]. CAP is considered severe when admission to the intensive care unit (ICU) is needed due to respiratory distress or septic shock and occurs in about 9–16 % of hospitalized patients [4, 5]. In patients with severe CAP, mortality rates ranged from 20 to 50 % according to the presence of shock, the accuracy of process of care, need for mechanical ventilation and underlying diseases [1–9]. Prompt initiation of appropriate antibiotic therapy and adequate resuscitation are recommended as it potentially benefits patients’ prognosis [10, 11] However, the mortality rate in immunocompetent patients admitted to the ICU by CAP with appropriate antibiotic therapy exceeds 20 % [3] suggesting that antibiotics alone are not enough.

Microvascular alterations are recognized as a key characteristic contributing to organ dysfunction and death in patients with sepsis [12–14]. Microvascular dysfunction leads to reduced oxygen delivery and extraction, causing heterogeneous and deficient tissue oxygenation, which is associated with adverse clinical outcome.

Global hemodynamic and metabolic parameters are used in current practice as resuscitation endpoints in severe sepsis and septic shock [11]. However, normalization of relevant variables such as mean arterial pressure (MAP), central venous pressure (CVP), cardiac output (CO) or serum lactate is not enough to define oxygenation status and aerobic metabolism in peripheral tissues [15–21] Increasing evidence suggests that regional oxygen saturation index (rSO_2_) determined by near-infrared spectroscopy (NIRS) might allow early detection of patients at risk of serious complications and have prognostic implications [17, 18].

We hypothesized that brachioradialis muscle rSO_2_ could reflect the prognosis in patients with severe CAP.

## Methods

### Ethics, consent and permissions

The study was performed from January 2011 to December 2013 in a 30-bed medical-surgical ICU in a tertiary university hospital. It was a prospective single centre, observational clinical study. The investigation was conducted according to the principles outlined in the Declaration of Helsinki. The study protocol was approved by the Joan XXIII University Hospital Ethics Committee (MICRO2 20/2010), and informed consent was given by each patient or their next of kin.

### Study population

Consecutive adult patients with severe CAP requiring admission to the ICU were enrolled. The diagnosis of CAP was based on (a) the detection of a new and persistent pulmonary infiltrate for which there is no other explanation and at least two of the following clinical criteria: (1) fever or hypothermia (temperature >38 or < 35.5 °C); (2) leucopenia or leukocytosis (white blood cells ≤4x10^9^/L^−1^ or ≥12 x10^9^/ L^−1^) or (3) purulent respiratory secretions and (b) acquisition of the infection outside a hospital, long-term care facility or nursing home [22]. Patients were admitted to the ICU if they presented at least one of following criteria: signs and symptoms of respiratory failure (respiratory rate >30/min, accessory musculature utilization, low SpO_2_ despite oxygen supplementation), needed mechanical ventilation, or presented with criteria of severe sepsis or septic shock. All patients admitted to the ICU were considered to present severe CAP. We decided to include only CAP patients in order to obtain a homogeneous population of patients to increase the internal validity of the study.

We excluded (a) patients <18 years old; (b) immunosuppression, defined as any primary immunodeficiency or immunodeficiency secondary to HIV infection, active malignancies, radiation treatment or use of cytotoxic drugs, or steroids drugs (daily doses >40 mg of prednisolone or equivalent for >2 weeks), immunological disease, solid organ transplant and haematological disease; (c) hospital-acquired pneumonia or health-care-associated pneumonia; (d) morbid obesity (body mass index >30 kg/m^2^); (e) clinical edema and (f) injuries in forearms.

### Study protocol

Patients were enrolled at ICU admission. Information collected included demographic characteristics, Acute Physiology and Chronic Health Evaluation (APACHE II) score [23], Sequential Organ Failure Assessment (SOFA) score [24] and global hemodynamic variables as heart rate (HR), CVP, MAP and mixed venous oxygen saturation (SvO_2_) when possible. Serum lactate and base deficit were obtained as markers of resuscitation and were determined at baseline and 24 h after ICU admission. The patients were treated with fluid administration and vasopressor therapy as required, according to the local guidelines adapted from Surviving Sepsis Campaign guidelines [11]. Patients were monitored at ICU admission using a central venous pressure (CVP) or echocardiography depending on the attending physician’s decision. Resuscitation was guided using CVP or echocardiography-derived parameters, and if advanced hemodynamic monitoring was needed, a pulmonary artery catheter was inserted. Fluid resuscitation was administered by fluid bolus challenge with crystalloids and/or artificial colloids, targeting a CVP 8–12 mmHg or if the patient showed >20 % collapsibility of the inferior cava vein during inspiration in ventilated patients (>50 % collapsibility in patients in spontaneous ventilation). When fluid administration was not enough to improve the patient’s hemodynamic status, the vasopressor agent of choice was norepinephrine titrated to the 2 μg/kg/min maximum dose to maintain the MAP >65 mmHg. Shock was defined as the need for vasopressors for >4 h after fluid replacement [8]. In accordance with our local guidelines, all patients were treated with antibiotic combination therapy (ceftriaxone plus macrolide) for CAP. All patients were evaluated during the first 24 h after ICU admission. We recorded if, at the time of admission, patients met major criteria according to the American Thoracic Society (need of mechanical ventilation or shock) [25]. Treatment was administered by an independent physician team that was blinded for rSO_2_ values. Patients were followed up for outcome data until ICU discharge or ICU death.

### Regional oxygen saturation index (rSO_2_) measurements

A probe somasensor was placed on the medial forearm (brachioradialis muscle) at a distance 5 cm distal to the elbow of each subject to obtain skeletal muscle rSO_2_ measurement as described elsewhere [17, 18]. Measurements were obtained using a commercially available NIRS spectrometry system INVOS 5100C oximeter (Somanetics Corporation, Troy. MI, USA), with a non-sterile and disposable skin surface probe. This system functions with two NIRS probes, one with 30-mm and the other with 40-mm spacing between NIR light send and receive optical fibre tips. The 30-mm signal is subtracted from the 40-mm signal, with the intention of subtracting the skin and subcutaneous fat layer artefact from the underlying skeletal muscle. For all patients, rSO_2_ was recorded at baseline and at 24 h. NIRS data was not used in patients’ management. According to our previous data [17, 18], we defined a threshold in <60 % as a “low rSO_2_”.

### Primary and secondary outcomes

The main objective of this study was to determine the relationship between early brachioradialis rSO_2_ values and mortality. Secondary outcomes were to compare the discrimination power for ICU mortality of brachioradialis rSO_2_ with standard variables of resuscitation and to evaluate the patients’ evolution according to the variation of rSO_2_ in the first 24 h.

### Statistical analysis

Discrete variables are expressed as counts (percentage) and continuous variables as means and standard deviations or medians within the 25th to 75th interquartile range (IQR). For demographic and clinical characteristics of the patients, differences between groups were assessed using the chi-square test and Fisher’s exact test for categorical variables and the Student *t* test, Mann-Whitney *U* test or Kruskal-Wallis test for continuous variables. Pearson’s correlation coefficient was used to assess the association between continuous variables. The concordance of the values obtained for different correlations was assessed using the intraclass correlation coefficient (ICC), based on the model of analysis of variance for repeated measures by the process reliability. Cumulative survival was assessed using Kaplan-Meier plot. Cox regression analysis was performed to determine which variables were independently associated with mortality. We included the variables that showed a significant association with mortality in the univariate analysis as covariables in the model: APACHE II score, rSO_2_ value, presence of shock and need for mechanical ventilation. The predictive values for skeletal rSO_2_ and the other variables of resuscitation were calculated using a receiver operator characteristic (ROC) curve, and the area under ROC curve (AUROC) was computed. The ROC graph was a plot of all the sensibility/specificity pairs resulting from continuously varying the decision threshold over the entire range of results observed. Data analysis was made using SPSS for Windows 13.0 (SPSS, Chicago, IL, USA). For all analyses, *p* < 0.05 was considered significant.

## Results

Forty patients with severe CAP were enrolled. Median APACHE II score and SOFA score were 17.0 (IQR = 12–21) and 4.0 (IQR = 3–5) points respectively, with an overall ICU mortality of 20 %. Additional demographics, baseline and 24 h hemodynamic and biochemical data with simultaneous measurements of skeletal muscle rSO_2_ are shown in Table 1.

**Table 1:** Clinical characteristics, hemodynamic variables and biochemical data with simultaneous measurements of skeletal muscle rSO_2_ of 40 patients included

Variable	Overall (*n* = 40)	Survivors (*n* = 32)	Non-survivors (*n* = 8)
Demographic data			
Age (years), mean (SD)	55.5 (15.9)	53 (16.4)	65 (9.8)*
Male, *n* (%)	27 (67.5)	21 (65.6)	6 (75.0)
Severity of illness			
APACHE II score at day 1, median (IQR 25–75)	17 (12–21)	16 (12–20)	22 (18–34)**
SOFA score at day 1, median (IQR 25–75)	4 (3–5)	4 (3–5)	4 (3–8)
Major ATS criteria, median (IQR 25–75)	28 (70)	20 (62.5)	8 (100)**
Invasive mechanical ventilation, *n* (%)	21 (52.5)	13 (40.6)	8 (100)***
Severe sepsis, *n* (%)	18 (44.5)	17 (53.1)	1 (12.5)
Septic shock, *n* (%)	22 (55.5)	15 (46.9)	7 (87.5)*
Mean comorbidities, *n* (%)			
Tobacco use	3 (8.6)	3 (10.7)	0
Chronic obstructive pulmonary disease	12 (33.3)	10 (34.3)	2 (28.6)
Cardiac disease	8 (20)	6 (18.7)	2 (28.6)
Diabetes mellitus	7 (20)	5 (17.9)	2 (28.6)
Severe liver disease	1 (2.9)	0	1 (14.3)
Hemodynamic data, median (IQR 25–75)			
Heart rate, b/min	105 (86–114)	105 (87–114)	105 (85–113)
Heart rate at 24 h	90 (77–100)	92 (79–103)	85 (70–99)
Mean arterial pressure, mmHg	79 (71–94)	78 (72–98)	80 (69–88)
Mean arterial pressure at 24 h	79 (70–93)	76 (70–96)	79 (71–90)
Central venous pressure, mmHg	11 (9–15)	11 (8–15)	11 (10–14)
Central venous pressure at 24 h	13 (13–16)	11 (7–16)	14 (12–15)
Mixed venous oxygen saturation, %^a^	76 (70–79)	76 (69–79)	74 (74)
Mixed venous oxygen saturation at 24 h^a^	70 (68–83)	70 (68–80)	80 (70–91)
Biochemical data, mean (SD)			
Baseline serum lactate, mM/L	3.0 (3.1)	2.5 (1.6)	4.8 (6.1)
Serum lactate at 24 h	2.0 (2.2)	1.4 (0.6)	3.9 (4.0)
Baseline base deficit	−3.5 (5.2)	−3.6 (5.4)	−2.7 (4.3)
Base deficit at 24 h	−2.3 (4.1)	−1.9 (4.1)	−3.4 (4.2)
Baseline haemoglobin levels, mg %	11.9 (1.8)	12.1 (1.9)	11.2 (1.1)
Haemoglobin levels at 24 h	10.6 (1.7)	10.6 (1.9)	10.4 (1.1)
Baseline serum glucose, mg %	166 (74.7)	158 (75.3)	197 (66.8)
Serum glucose at 24 h	152 (75.6)	144 (79.4)	177 (58.7)
Baseline arterial pH	7.34 (0.1)	7.40 (0.1)	7.34 (0.3)
Arterial pH at 24 h	7.36 (0.07)	7.30 (0.07)	7.32 (0.1)
Baseline serum creatinine, mg %	1.40 (0.9)	1.38 (1.03)	1.41 (0.8)
Serum creatinine at 24 h	1.1 (0.59)	0.9 (0.4)	1.5 (0.9)
rSO_2_ brachioradialsis			
Baseline rSO_2_, mean (SD)	62.9 (10.7)	68 (61–72)	46 (43–57)***
rSO_2_ at 24 h, mean (SD)	65.4 (12.3)	69 (60–76)	52 (41–57)***
Baseline rSO_2_ < 60, *n* (%)	14 (35)	7 (21.9)	7 (87.5)***
rSO_2_ < 60 at 24 h, *n* (%)	6 (15.8)	4 (12.5)	7 (87.5)***
Therapy, mean (SD)			
Norepinephrine at baseline μg/kg/min	0.35 (0.26)	0.40 (0.14)	0.29 (0.36)
Norepinephrine at 24 h, μg/kg/min	0.35 (0.29)	0.22 (0.28)	0.52 (0.23)*
Fluid balance at 24 h, mL	1650 (1350)	1470 (800)	2300 (2500)
ICU length of stay			
Median IQR 25–75 %	18 (11–30)	18 (11–31)	24 (8–30)

*SD* standard deviation, *IQR* interquartile range, *APACHE II* Acute Physiology and Chronic Health Evaluation, *SOFA* Sequential Organ Failure Assessment, *ATS* American Thoracic Society, *rSO*_*2*_ regional oxygen saturation index

For survivors vs. non-survivors comparison *p = 0.01; **p < 0.05; ***p < 0.001

^a^Only 11 patients

Non-survivors had higher APACHE II score, greater need for invasive mechanical ventilation and higher frequency of septic shock compared to survivors. All non-survivors had major ATS criteria.

We found no significant differences in hemodynamic and biochemical data according to evolution. Brachioradialis rSO_2_ was the only variable associated with mortality at admission and at 24 h. Twenty-two patients (55 %) had shock at admission. Shock patients had lower rSO_2_ at baseline (59 % [SD 10.7] vs. 67 % [SD 8.9], *p* < 0.001) and at 24 h (61 % [SD 11.5] vs. 72 % [SD 10.6], *p* < 0.009) than patients without shock. However, we observed no significant correlation neither between MAP and rSO_2_ at baseline (*r* = 0.19, *p* = 0.90) and at 24 h (*r* = 0.07, *p* = 0.68) nor with rSO_2_ and norepinephrine doses at baseline (*r* = 0.11, *p* = 0.67) and at 24 h (*r* = −0.46, *p* = 0.06). In contrast, serum lactate at 24 h showed a weak but significant correlation with rSO_2_ at 24 h (*r* = 0.66, *p* = 0.001). In all cases, the concordance was poor and it was not significant. A central venous catheter for CVP measurement was inserted in 22 patients, the rest of them were monitored using echocardiography-derived parameters. SvO_2_ was available in only 11 (27, 5 %) patients. SvO_2_ values were 76 and 70 % at baseline and at 24 h, respectively. We observed no significant correlation between rSO_2_ and SvO_2_ at any time of the study (data not shown).

**Fig. 1:**
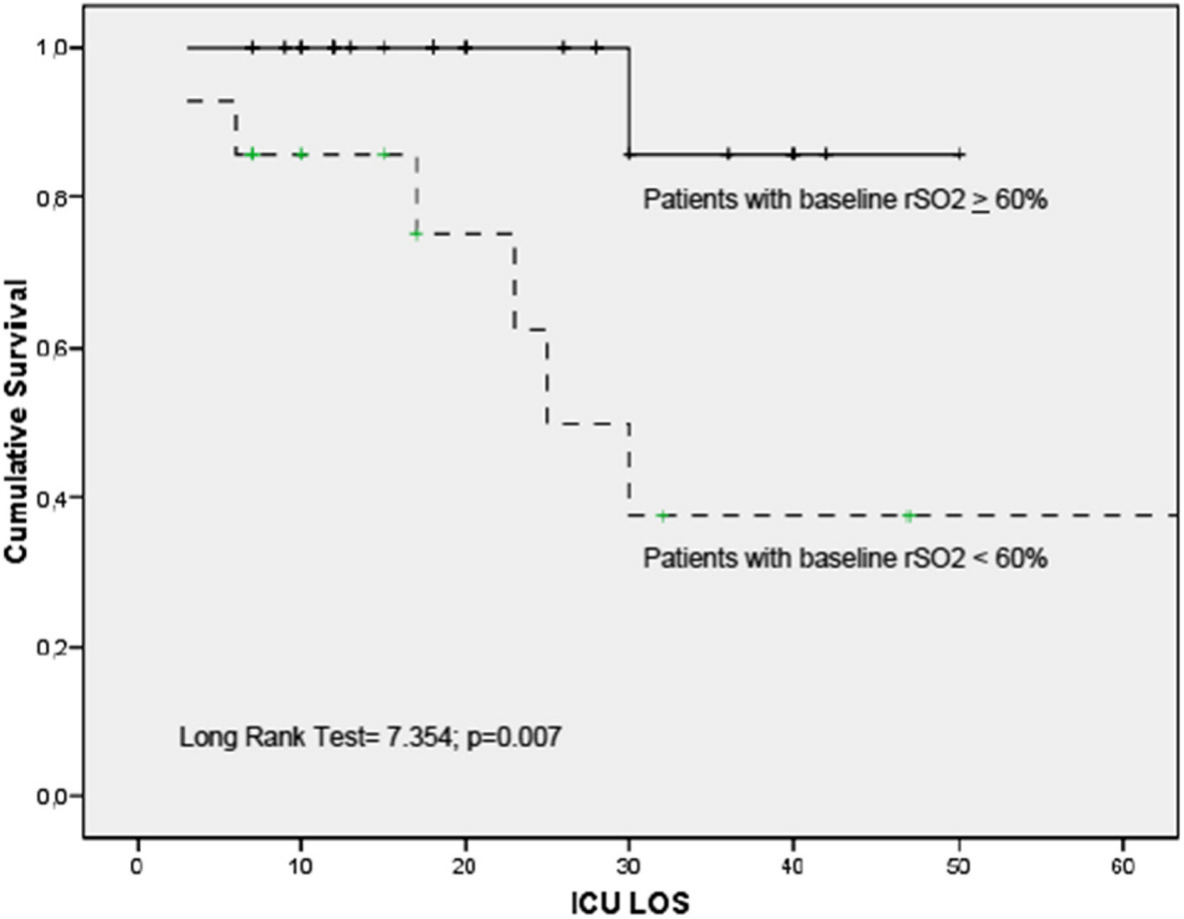
Survival graph (Kaplan-Meier plot) for patients according baseline rSO_2_ cut-off values. *ICU LOS* intensive care unit length of stay (days), *rSO*_*2*_ regional oxygen saturation index

**Fig. 2:**
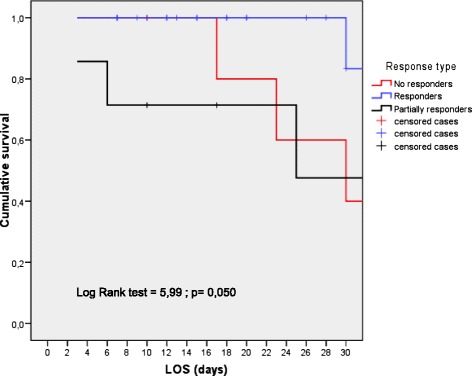
Survival graph (Kaplan-Meier plot) for patients according to variation (0–24 h) of rSO_2_ with treatment. No responders: which showed no improvement or a decrease in rSO2 value; Responders: patients which showed an improvement in rSO2 value to reach rSO2 of >60 % at 24 h and; Partially responders: patients in which the rSO2 value improved but did not reach >60 % at 24 h; *ICU LOS* intensive care unit length of stay (days), *rSO*_*2*_ regional oxygen saturation index

Patients with baseline “low rSO_2_” had a significantly higher ICU mortality rate than patients with rSO_2_ ≥ 60 % (Fig. 1). Fourteen patients (35 %) had a baseline skeletal muscle rSO_2_ < 60 %, and seven of them (50 %) died. In contrast, only one patient (3.8 %) with rSO_2_ ≥ 60 % died (*p* = 0.001). This represents more than sixfold increase in the risk of death (OR 6, 25; 95 % CI 1.0–39.4) despite similar levels of severity of illness and resuscitation variables (Table 2). We differentiate patients according to variation of the value of rSO_2_ from ICU admission to 24 h later. Three types of trends were observed: (1) a group of patients which showed no improvement or a decrease in rSO_2_ value (no responders); (2) other group which showed an improvement in rSO_2_ value to reach rSO_2_ of >60 % at 24 h (responders); and (3) a group of patients in which the rSO_2_ value improved but did not reach >60 % at 24 h (partially responders). We observed different outcomes according to initial rSO_2_ variation with a higher mortality rate in “no responders” (*n* = 3/7, 42.9 %) and “partially responders” (*n* = 3/7, 42.9 %) patients than “responder patients” (*n* = 1/26, 3.8 %, *p* < 0.005) with an OR = 18.7, 95 % CI 1.17–625.6 and a log rank = 5.99, *p* = 0.05 (Fig. 2). In addition, we performed a Cox regression analysis to determine if rSO_2_ < 60 % was associated with mortality. The regression analysis showed that “low” rSO_2_ at ICU admission (hazard ratio (HR) = 8.99; 95 % CI 1.05–76.8; *p* = 0.045) and “low” rSO_2_ at 24 h (HR = 13.18; 95 % CI 1.52–113.6; *p* = 0.019) were variables independently associated with mortality. In contrast, APACHE II score (HR = 1.09; 95 % CI 0.99–1.19; *p* = 0,052) was not associated with mortality (Table 3).

**Table 2:** Levels of severity of illness and baseline resuscitation variables in 40 patients with severe community-acquired pneumonia according to basal brachioradialis muscle rSO_2_

Variable	rSO_2_ < 60 % (*n* = 14)	rSO_2_ ≥ 60 % (*n* = 26)	*p* value
APACHE II score , median (IQR 25–75)	18 (15–22)	15 (12–22)	0,26
SOFA score, median (IQR 25–75)	4,5 (3–5,5)	4 (3–5)	0,73
MAP (mmHg), median (IQR 25–75)	84 (69,7–93)	75 (71,7–94,7)	0,64
SL (mM/L), mean (SD)	4,2 (4,8)	2,3 (1,5)	0,17
BD, mean (SD)	−1,7 (6,7)	−4,4 (3,9)	0,11
Catecholamines (μg/kg/min), mean (SD)	0,39 (1,5)	0,32 (0,32)	0,66
Fluid balance at 24 h (mL), mean (SD)	2130 (2200)	1450 (759)	0.18
Need for MV, *n* (%)	8 (57)	13 (50)	0,58
ICU mortality rate, *n* (%)	7 (50)	1 (3,8)	0,001

*APACHE II* Acute Physiology and Chronic Health Evaluation, *SOFA* Sequential Organ Failure Assessment, *MAP* medium arterial pressure, *SL* serum lactate, *BD* base deficit, *rSO*_*2*_ regional oxygen saturation index, *MV* mechanical ventilation, *IQR* interquartile range, *SD* standard deviation

**Table 3:** Results of cox regression analysis

Variable	Hazard ratio	95 % CI	*p* value
Presence of shock	1.01	0.09–11.15	0.98
Need of MV	29.8	0.01–4.2E	0.94
APACHE II	1.09	0.99–1.19	0.052
“low” rSO_2_ (admission)	8.99	1.05–76.8	0.045
“low” rSO_2_ (24 h)	13.18	1.52–113.6	0.019

*MV* mechanical ventilation, *APACHE II* Acute Physiology and Chronic Health Evaluation, *rSO*_*2*_ regional oxygen saturation, *CI* confidence interval

Finally, the discriminatory ability of each variable for ICU mortality was measured using the area under ROC curves (AUC). The AUC showed consistent mortality discrimination at baseline (0.84; 95 % CI 0.626–1.057, *p* = 0.004) and at 24 h (0.86; 95 % CI 0.646–1.075, *p* = 0.002) for rSO_2_ values, better than other resuscitation variables (Table 4).

**Table 4:** Area under ROC curve (AUROC). Discrimination for mortality of rSO_2_ and hemodynamic variables and APACHE II score

Variable	AUROC	95 % CI	*p* value
rSO_2_ baseline	0.84	0.626–1.057	0.004
24 h	0.86	0.646–1.075	0.002
Lactate baseline	0.57	0.334–0.820	0.51
24 h	0.69	0.434–0.951	0.10
BD baseline	0.44	0.200–0.680	0.61
24 h	0.70	0.477–0.927	0.08
MAP baseline	0.58	0.391–0.782	0.46
24 h	0.52	0.321–0.726	0.83
APACHE II	0.76	0.559–0.980	0.02

*CI* confidence interval, *rSO*_*2*_ regional oxygen saturation index, *BD* base deficit, *MAP* medium arterial pressure, *APACHE II* Acute Physiology and Chronic Health Evaluation

## Discussion

The main finding of our study is that in a population of patients with severe CAP, an early value of skeletal muscle rSO_2_ <60 % was associated with a worse prognosis despite similar severity of illness, representing near sixfold increase in the risk of death compared with patients with rSO_2_ ≥ 60 %. In addition, rSO_2_ variation (from admission to 24 h) allowed distinguishing three different types of responses to treatment which were associated with different evolution. To the best of our knowledge, our data is the first to report the relationship between rSO_2_ alterations and CAP prognosis with this NIRS device.

Despite advances in diagnosis and antimicrobial therapy, severe CAP remains an important cause of morbidity and mortality, especially in patients requiring ICU admission [1–6]. Unfortunately, it remains unknown whether specific clinical conditions or biomarkers can be used to identify patients with potential high risk of death.

Several risk factors that predict which patients will develop moderate to severe CAP have been identified in a small number of studies [1–3]. Our group [26] found that a prompt oxygenation assessment in the emergency department shortened the time taken to initiate antibiotic therapy and improved survival. Practice guidelines based on severity assessment tools, such as the ATS admission criteria or CURB-65 score, allow high-risk patients to be identified and given specific treatment. However, the prediction rule is derived from clinical data and laboratory parameters which are time-consuming, thereby limiting the clinical applications of predictive outcomes [27, 28]. Several biomarkers have been proposed to assess severity of illness and outcome. Inflammatory cytokines such as interleukin-6, procalcitonin, albumin and C-reactive protein levels have also shown to predict the severity of illness and 28-day mortality [29, 30]. However, most of these factors merely reflect individual coagulation and inflammation status and have no therapeutic potential in themselves. Therefore, identification of variables capable of not only predicting outcomes but also providing a potential therapeutic target would be more useful in clinical applications.

Microvascular alterations are frequently observed in patients with sepsis. In humans, several studies have shown that severity of the alterations in microvascular perfusion is associated with poor outcome [12–14]. A crucial question is whether these microcirculatory alterations merely reflect the severity of the disease or contribute independently to mortality. Among the tools available for tissue microcirculation/oxygenation assessment, NIRS devices seem promising. It has been shown in different life-threatening conditions that muscle skeletal oxygen saturation might characterize tissue hypoperfusion and effectiveness of therapy in trauma [20] and septic shock [17–19, 21]. However, the relationship between skeletal oxygen saturation with macrohemodynamics variables is not entirely clear. Small-sized trials showed that microvascular alterations were relatively independent of MAP, CO and vasopressor agent use [19, 31, 32]. In our study, no significant correlation was observed between the values of NIRS and classic hemodynamic variables. In contrast, other authors observed a significant relationship between CO and skeletal muscle oxygen saturation [20, 33]. In this way, our data cannot be compared with that available in the literature using other NIRS devices. Important technical differences should be considered before interpreting and comparing data based on NIRS technology. In the near-infrared range, oxyhaemoglobin (HbO_2_), deoxyhaeamoglobin (Hb) and oxidized cytochrome oxidase (CytOx) have characteristic absorption spectra. In order to derive concentration changes simultaneously for Hb, HbO_2_ and CytOx, values for absorption at four wavelengths of near infrared are often used. The calculation used to solve the modified Beer-Lambert equation at each of these wavelengths is known as an algorithm. Algorithms are different for each device and also vary depending on the presence of other chromophores and the precise values for the absorption coefficients chosen [34–36]. Recent work has applied different published algorithms to the same data set and revealed striking differences in the calculated concentration changes [37, 38]. In addition, it is crucial to know the differential pathlength factor (DPF) when interpreting NIRS data. DPF is the distance travelled by each photon, and its value is derived from studies in healthy adults [39]; it may vary in other situations and is also wavelength-dependent. DPF may also change within the same subject over a period of time if the state of the tissue or tissue geometry alters [40]. Thus, probably, “normal” and critically “abnormal” tissue oxygenation values should be determined according to the critically ill patient population and the NIRS device used.

Besides differences regarding technical characteristics of each NIRS device, different values have been obtained when measuring rSO_2_ in different muscles. Our group found different values of rSO_2_ in brachioradialis and deltoid muscles of septic patients, although in each location “low” rSO_2_ values were associated with poor outcome [18]. Other groups have found these differences between muscles [41].

Although our patients in shock had lower values of rSO_2_ during the entire study period, we observed a non-significant relationship between MAP, vasopressor dose administered or SvO_2_ and rSO_2_. These findings may suggest that these variables do not affect rSO_2_ significantly. Classically, in septic shock, adequacy of perfusion for oxygen demand is assessed by serum lactate and base deficit levels [11, 15]. In the present study, serum lactate and base deficit levels were not associated with poor outcome. Skeletal muscle rSO_2_ may provide a more accurate reflection of oxygen delivery because it represents the balance between the oxygen supply to the capillaries directly beneath the sensor and oxygen consumption at that site independently of global hemodynamic state [17]. The rSO_2_ is indicative of oxygen extraction from haemoglobin and in comparison with arterial oxygen saturation, and possibly SvO_2_, it might provide a more accurate picture of hypoxia [17]. In fact, according to present data, the microcirculatory response to the treatment administered might be evaluated by the variation of rSO_2_. Patients with basal “low” rSO_2_ that respond appropriately to early treatment (first 24 h) and normalize the rSO_2_ (≥60 %) presented an ICU evolution significantly better than patients who despite improving rSO_2_ did not reach this cut-off.

The discriminatory ability for mortality was substantially higher for rSO_2_ than serum lactate and base deficit (except for 24 h). This suggests that skeletal muscle rSO_2_ can detect early poor tissue oxygenation that results in serum lactate or base deficit elevation during septic shock. Taking these into account, it is reasonable to think that rSO_2_ emerges as an early indicator of poor oxygenation with implications on prognosis. In addition, Cox regression analysis showed that “low” rSO_2_ value, both at ICU admission and at 24 h, was independently associated with mortality.

Our study had several limitations. First, the sample size was small in a single centre, which may account for some lack of statistical significance. The small patient population also prevented a subgroup analysis to further examine the differences in the effects of rSO_2_ on clinical outcomes according to some special clinical condition. However, clear differences in rSO_2_ values were observed between survivors and non-survivors. This suggests that rSO_2_ gives early evidence of patients with severe CAP who suffer oxygenation alterations with prognostic implications. Second, we not have been able to calculate the sample size to be included because there is no previous data with this population of patients and this NIRS device. However, we calculate the statistical power of our study which has been very high. Accepting a risk alpha of 0.05 in a bilateral contrast with 26 subjects in the first group (≥60 %) and 14 in the second (<60 %), the power of the contrast of hypotheses is 94 % to detect as a statistically significant difference that exists between 3, 5 % in the first group and 50 % in the second for mortality.

Third, NIRS does not directly measure microcirculatory flow. However, many studies [42, 43] have observed that NIRS values correlated well with global and specific organ perfusion parameters. In addition, NIRS signal is limited to vessels that have a diameter <1 mm (arterioles, capillaries and venules) and may be a useful tool for non-invasive monitoring of microcirculation in septic patients [13]. Fourth, we evaluated ICU mortality and not longer-term mortality, but the latter may also be influenced by other factors that are more related to the underlying disease and comorbidities. Fifth, we only included patients with CAP for which our data cannot be translated to other populations of patients with sepsis. Finally, we did not carry out a vascular occlusion test (VOT). VOT might to improve and expand the predictive ability of rSO_2_ in several scenarios [19, 44, 45]. However, our findings showed a high discriminatory power for absolute rSO_2_ values in accordance with our previous findings [17].

## Conclusions

Our findings suggest that forearm skeletal muscle rSO_2_ determined at an early stage of severe community-acquired pneumonia is likely to be associated with outcome. Future studies are required to further substantiate our findings and to confirm the potential benefits of assessing skeletal muscle rSO_2_ on severe CAP.
